# Supplementary material for: Analyzing and forecasting under-5 mortality trends in Bangladesh using machine learning techniques
Source: PLoS One. 2025 Feb 7;20(2):e0317715. doi: 10.1371/journal.pone.0317715 (PMC11805350; doi:10.1371/journal.pone.0317715)
Supplement: S1 Data — (DOCX) [file pone.0317715.s001.docx]

| **Survey Name** | **Under 5 Mortality rate (per 1,000 live births)** |
| --- | --- |
| BDHS 1993-94 | 134 |
| BDHS 1996-97 | 116 |
| BDHS 1999-00 | 94 |
| BDHS 2004 | 88 |
| BDHS 2007 | 65 |
| BDHS 2011 | 53 |
| BDHS 2014 | 46 |
| BDHS 2017-18 | 45 |

Data Source [https://dhsprogram.com/data/available-datasets.cfm](https://dhsprogram.com/data/available-datasets.cfm).
