# Supplementary material for: Analyzing and forecasting under-5 mortality trends in Bangladesh using machine learning techniques
Source: PLoS One. 2025 Feb 7;20(2):e0317715. doi: 10.1371/journal.pone.0317715 (PMC11805350; doi:10.1371/journal.pone.0317715)
Supplement: Attachment — Submitted filename: point by point Response - Review 1.doc [file pone.0317715.s003.doc]

**Response to Editors & Reviewers**

***“Analyzing and Forecasting Under-5 Mortality Trends in Bangladesh using Machine Learning Techniques”***

**Ref: Manuscript Number:PONE-D-24-41552- [EMID:98e0e853d61e585b]**

Dear Editors,

Regarding our manuscript, we thank you for the comments sent on October 22, 2024. My co-authors and I have revised the manuscript accordingly and would like it to be reconsidered for publication. As requested, we have included point-by-point responses to editors’ and reviewers’ comments below.

Please let us know if anything further is required at this time, and we thank you very much for considering our revised manuscript.

Shayla Naznin

| **Editors comments** | **Response** |
| --- | --- |
| The paper has limitations in terms of its technical contents and presentation.  Please address all the comments by the reviewers carefully in your revised submission. Thank you. | Thank you very much for your feedback! We have endeavored to address all of the reviewers’ comments as detailed below to further strengthen the paper. |
| **Reviewer 1** |  |
| 1.The author should explore the distribution characteristics of data and the correlation between variables, so as to better explain why the linear model performs best. | Thank you for this careful reading of our Abstract has been revised line#210-218 |
| 2.Why does 5 - fold cross - validation perform best in most models? The author can analyze the distribution characteristics of data, the relationship between the sample quantity and the number of folds. | The superior performance of 5-fold cross-validation likely arises from its balance between bias and variance, particularly with the dataset's small size. Splitting the data into 5 parts ensures adequate training data in each fold while maintaining reasonably sized validation sets, avoiding the instability of higher folds (e.g., leave-one-out) or the inefficiency of fewer folds. This balance makes it well-suited for small datasets. |
| 3.The research uses BDHS data and mentions integrating supplementary data from the World Bank and UNICEF. It is necessary to explain how these supplementary data enhance the robustness of the analysis. | Thank you for this careful reading this has been revised kindly address the line #131-164 |
| 4.The annual reduction rate is simply presented, but how ARR changes over time has not been explored. The connection between the change of ARR and specific intervention measures should be discussed. | Thank you for this careful reading this has been revised in the line #427-440 |
| 5.The research predicts the trend until 2035, but does not discuss the reliability of long - term prediction, which is not rigorous enough. | Thank you for this careful reading this has been revised kindly address the line #525-529 |
| **Reviewer 2** |  |
| Comment #1 - (Abstract) - Linear Regression Overemphasis The abstract focuses too much on Linear Regression, neglecting a balanced discussion of other models' performance. | Thank you for this careful reading this has been revised kindly address the line #10-63 |
| Comment #2 - (Introduction) - Lack of Machine Learning Rationale The introduction does not explain why ML techniques were chosen over traditional methods. | Thank you for this careful reading this has been revised kindly address the line #67-130 |
| Comment #3 - (Methods) - Missing Model Justification No rationale is provided for selecting specific ML models like CatBoost or XGBoost. | Thank you for this careful reading this has been revised kindly address the line #131-251 |
| Comment #4 - (Methods) - Insufficient Data Preprocessing Details The paper lacks detail on how missing data and outliers were handled during preprocessing. | Thank you for this careful reading this has been revised kindly address the line #131-251 |
| Comment #5 - (Results) - Linear Regression Focus Other models' poor performance is not adequately discussed, with an overemphasis on Linear Regression. | Thank you for this careful reading this has been revised kindly address the line #252-447 |
| Comment #6 - (Results) - No External Validation The study lacks external validation to confirm the models' forecasting accuracy. | Thank you for this careful reading this has been revised kindly address the line #252-447 |
| Comment #7 - (Discussion) - Lack of Policy Implications The discussion does not translate the findings into specific policy recommendations. | Thank you for this careful reading this has been revised kindly address the line #449-506 |
| Comment #8 - (Discussion) - Missing External Variables Important variables like healthcare access and maternal health are not included in the models. | Thank you for this careful reading this has been revised kindly address the line #449-506 |
| Comment #9 - (Limitations) - Insufficient Data Bias Acknowledgment The limitations section does not sufficiently address biases in the self-reported data. | Thank you for this careful reading this has been revised kindly address the line #508-543 |
| Comment #10 - (Conclusion) - Overly Optimistic Projections The projections may be too optimistic, without considering potential socio-economic changes. | Thank you for this careful reading this has been revised kindly address the line #545-597 |
| **Reviewer 3** |  |
| Abstract:  1. Conciseness: The abstract is comprehensive but could be slightly condensed for better readability. Consider trimming redundant phrases without losing essential details.  - For example: “employs advanced machine learning models including...” could be shortened to “employs machine learning models like...” | Thank you for this careful reading this has been revised kindly address the line #10-63 |
| 2. Impact Statement: Add a brief sentence at the end highlighting the potential impact of your findings on policy and health interventions in Bangladesh. | Thank you for this careful reading this has been revised kindly address the line #10-63 |
| Background:  1. Context Clarity: Provide a brief explanation of why machine learning models are preferable for forecasting under-5 mortality, perhaps elaborating on the limitations of traditional methods.  2. Relevance: Emphasize the significance of choosing Bangladesh for this study, and how findings here may relate to global child mortality trends in similar countries. | Thank you for this careful reading this has been revised kindly address the line #10-63 & 67-130 |
| Methods:  1. Model Justification: Explain briefly why so many machine learning models were tested. It would be useful to state why linear models performed better for this dataset, such as due to linear trends in mortality rates.  2. Error Metrics: The evaluation metrics used (MAE, RMSE, etc.) are appropriate, but you could explain why these specific metrics were chosen and how they relate to practical applications of the forecasts.  3. Temporal Variables: When discussing lag features and rolling averages, briefly explain why these were incorporated to improve the model. | Thank you for this careful reading this has been revised kindly address the line #131-251 |
| Results:  1. Model Discussion: More emphasis could be placed on why linear models performed significantly better than non-inear models (e.g., Decision Tree, XGBoost). You could hypothesize why non-linear relationships were not as critical in predicting mortality rates.  2. Table Presentation: The table comparing model performances is very informative, but ensure the table is well-labeled with clear headings and definitions for each metric (e.g., explain MAE briefly).  3. Interpretation: In discussing the accuracy metrics, try to make a stronger connection between the model performance and real-world implications (e.g., how a lower RMSE can translate to better policy planning). | Thank you for this careful reading this has been revised kindly address the line #252-447 |
| Conclusions:  1. Policy Implications: While the conclusion mentions the gap between forecasted values and the SDG target, further elaboration on how the predictions can influence specific interventions or where further resources should be directed would strengthen the section.  2. Future Work: Suggest potential ways to improve the forecasting models or factors that future research should consider (e.g., incorporating more complex social or environmental variables). | Thank you for this careful reading this has been revised kindly address the line #545-597 |
| General Suggestions:  - Grammar/Flow: Some sentences could be streamlined for better readability. For instance: “These techniques offer improved accuracy and reliability in forecasting providing valuable insights...” could be rephrased for smoother flow.  - Consistency: Ensure consistent use of terms like “under-5 mortality” and “U5M” throughout the article.  - Visuals: Including a well-labeled graph or chart illustrating the predicted trend in under-5 mortality over time would make the results more digestible for the reader.  Overall, the article is informative and presents the data in a clear, structured manner. With a few adjustments to clarity, flow, and further explanation of key points, it will be more impactful. | Thank you for this careful reading this has been revised |
